# Chicago Medical Society, July 20th

**Published:** 1885-08

**Authors:** 


CHICAGO MEDICAL SOCIETY.

*Stated Meeting, July 20, 1885,* S. J. JONES, Chairman, *pro tem.*

PROFESSOR W. E. CASSELBERRY read an interesting paper detailing the surgical procedures in a case of membranous occlusion of the posterior nares. His patient was aged about fifty, a native of Russian Poland, and had suffered from obstruction of the left nasal chamber for the last thirteen years. Thick, viscid and foul muco-purulent crusts accumulated in the nares and naso-pharyngeal space which he could neither expectorate nor expel through the nose on account of the impenetrability of the nostril to currents of air. Partial occlusion, also, of the right nasal chamber necessitated frequent and prolonged mouth-breathing, and consequently he has suffered from atrophic pharyngitis and laryngitis, with painful deglutition, cough, suffocative paroxysms, etc. Deafness in the left ear and annoying *tinnitus aurium* have long been prominent symptoms. Most violent paroxysmal headaches, constant soreness on the top of the head, vertigo, especially upon stooping, and various indescribable cephalic sensations of a most distressing character, have served to render life miserable.

By a rhinoscopic examination the pharynx was seen to be covered with a foul, viscid, muco-purulent substance, continuous in descent from the naso-pharyngeal space, and imparting to the expired air a disgustingly fetid odor. The naso-pharynx was filled with rotten crusts which necessitated removal for further inspection of the parts. Having thoroughly cleansed the parts and accustomed the patient to instrumental manipulation, by using Voltolini's uvula holder, a good rhinoscopic image was obtained. All previous efforts to pass a probe from in front had failed, and passing the finger behind the palate through the mouth, an obstruction was encountered. The rhinoscopic image revealed a tense membrane covering the left choana almost completely. Its free edge was thin and sharp and approached so near to the septum, that only a small slit or opening remained between it and the septum narium. The left ostium tubæ could not be seen, the membrane evidently lying behind the Eustachian orifice, and so intercepting its image. On passing a probe between the membrane and septum the edge of the membrane could be pushed backward. It felt tense and was from one to two millimeters in thickness.

The right choana was partially covered by a membrane extending half across the aperture and intercepting the view of the superior and outer half of the middle turbinated bodies, and the right ostium tubæ. This membrane was much thicker and less tense than that on the left side.

In order to prepare the patient for treatment, he was trained daily for one week in the introduction of mirrors and instruments. A flexible silver probe was bent, after many trials, to exactly the proper curve to pass readily by way of the mouth and naso-pharynx into the opening on the left side and to pick up the membrane. A straight knife-electrode was then made to conform to the same curve, and lastly, this instrument, with the attachments complete, made several trial trips to the desired locality. All being in readiness, December 13, 1884, the knife-electrode was introduced through the opening between the membrane and septum and its edge pressed backwards against the membrane. At this moment the patient retracted the soft palate and shut off all vision. But, thanks to previous training, when instructed to relax all the parts the velum fell and the rhinoscopic image was perfect. Having adjusted the electrode properly the current was turned on, the knife end became red hot in an instant and the membrane was incised without bleeding, and without sufficient pain to cause the patient to wince. In another instant the current was broken, the electrode cooled in a few seconds and was withdrawn. An examination revealed that the work had been wonderfully efficient. The membrane having been tense, when it was incised, each part retracted leaving an opening sufficiently large to bring into view the middle and inferior turbinated bodies, and to the delight of the patient to allow a free passage of air through the left nostril, and from that moment he has had none of the distressing cephalic symptoms mentioned. The floor of the nose, the septum and the turbinated bodies were thickly incrusted with horribly fetid, cheesy masses, decomposed and desicated secretions accumulated for years, which were removed with great difficulty. The catarrhal symptoms then gradually became less distressing. After two similar operations the left ostium tubæ became visible in the rhinoscopic mirror, and two or three touches with the knife served to entirely obliterate the membrane. Four similar operations served to obliterate the membrane in the right side, and now both posterior nares are widely patent and in an approximately natural condition.

It is probable that atresia of the posterior nares is by no means so rare as the few reported cases and the omission of all mention in many treatises might lead us to suppose. The reporter had seen reports of two cases only occuring in the adult, one by Voltolini and another by Pomeroy. Records of cases occuring in infancy are more common, doubtless because the nursing function necessitates either a correct diagnosis and operation for relief, or death ensues and an opportunity for a postmortem examination. Reports of such cases are recorded by Ronaldson, Luschka, Betts, Cohen and Emmerts. In all these cases the malformation was congenital and it was probably so in the case above reported. This patient has never suffered from any illness which would act as a cause for the development of such a membrane. It may be possible that in earlier years the membrane was relaxed and less complete, similar to the condition found on the right side, and later it underwent contraction, became thinner, more tense and sufficiently extended to cover the choana. The left membrane was composed chiefly of a reduplicature of mucous membrane, with possibly some connective tissue and muscular fibres interlying in the thicker portion toward the outer border. The right structure contained considerable muscular tissue. There was no osseous malformation. Upon the left, the attachments were, apparently, to the mucous lining of the superior posterior edge of the vomer, the inferior surface of the body of the sphenoid bone, the left pharyngeal wall behind the Eustachian orifice and the superior posterior surface of the velum palati. Upon the right the attachments included only the inferior surface of the body of the Sphenoid, and the right pharyngeal wall behind the Eustachian orifice.

The galvano-cautery is more useful in such operations because it is a dull instrument; can be introduced or withdrawn without harm ; can be used when applied to the proper place, and almost no bleeding or pain ensues.

The society adjourned after the paper was read and Professor Casselberry had exhibited a Fleming galvano-cautery battery with electrodes—as modified by Dr. Carl Seiler.

